# Raster-scanning Donut simplifies MINFLUX and provides alternative implement on other scanning-based microscopes

**DOI:** 10.1038/s41377-022-00983-6

**Published:** 2022-10-10

**Authors:** Xinzhu Xu, Shu Jia, Peng Xi

**Affiliations:** 1 Department of Biomedical Engineering, College of Future Technology, Peking University, Beijing, 100871 China; 2 Wallace H. Coulter Department of Biomedical Engineering, Georgia Institute of Technology and Emory University, Atlanta, Georgia 30332 USA; 3 UTS-SUStech Joint Research Centre for Bio-medical Materials & Devices, Department of Biomedical Engineering, College of Engineering, Southern University of Science and Technology, Shenzhen, Guangdong 518055 China; 4 National Biomedical Imaging Center, Peking University, Beijing, 100871 China

**Keywords:** Super-resolution microscopy, Biophotonics

## Abstract

A donut excitation moves around a single molecule with a zigzag configuration lattice by lattice. Such a method implemented in scanning fluorescence microscopy simplifies the conventional MINFLUX process. Consisting of hollow zero-intensity excitation, single-pixel detection, time-correlated single photon counting, and drift stabilization, the system achieves localization precision and resolution very close to conventional MINFLUX theoretically and experimentally. An averaged high-SNR reference, and pixel-registered intensity from a single molecule is essential to reconstruct localization in maximum likelihood estimation. With performance reaching nearly conventional MINFLUX’s, the proposed raster-scanning MINFLUX can inspire researchers expertized in STED or confocal setup to quickly transform to MINFLUX and develop for further exploring on bio-specimens or optical applications.

## Abstract (web summary)

Consisting of donut excitation, xy-scan, drift correction and time-related single photon detection modules, RASTMIN possesses high compatibility with scanning-based fluorescent microscope, if starting from the experience on STED construction.

## Graphical abstract

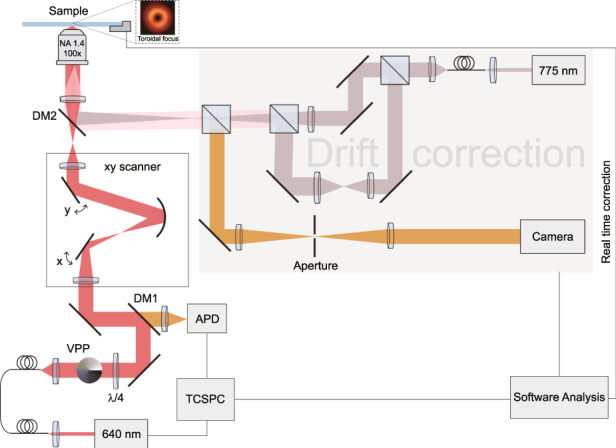

The advent of super-resolution fluorescence microscopy has opened up a promising avenue to the complete understanding of cell biology, from confocal fluorescence microscopy^1^, which promotes signal-to-noise ratio and thus resolution, to STED^2^ that precisely coincides with Gaussian excitation and donut depletion, and nonlinear SIM (N-SIM)^3^ inspired by the excited saturation contributing to Fourier domain extension or even PALM^4^ /STORM^5^ based on single-molecule localization. They have favored researchers to directly observe the subcellular organelle morphology (mitochondrial cristae^6^), the fine subcellular structure (microtubules^7,8^, neurons skeleton^9^ and active zone^10^), and further boosted the qualitative leap in the understanding of complex life science process (heterogeneity and dynamics of membranes^11^ and even the first-observed enlarged fusion pores during vesicle exocytosis^12^).

In the recent lustrum, taking advantage of the characteristic of the central zero-intensity of donut excitation and the statistical estimation, Balzarotti developed MINFLUX^13^ in 2017, and Gwosch published 3D-MINFLUX^14^ in 2020. These techniques have boosted the localization precision of optical fluorescence microscopy to ~1 nm, with the corresponding spatial resolution of ~1–3 nm and temporal resolution of ~50 μs. However, the methods that achieve such ultra-precise localization precision and resolution require complicated instruments such as ultra-fast optical scanning devices and delicate FPGA circuits, which inevitably leads to a significant increase in system complexity, technical barriers, and instrumentation cost. This has been a bottleneck for other laboratories to continue to develop and innovate based on the MINFLUX system, thus posing a challenge for its broad applicability. Building a more straightforward alternative system with comparable performance is one of the issues that need attention in the super-resolution fluorescence microscopy field. In 2021, the pulsed MINFLUX^15^ invented by Luciano et al. promised a turning point to this problem. The excitation, consisting of four parallelly spaced and equal-interval pulsed lasers, is built on an existing point-scanning confocal microscopy system. In combination with MINFLUX’s four-point targeted coordinate pattern (TCP) excitation, scanning and time-correlated single-photon detection module, they achieved a localization precision of 1~2 nm under 1000–2000 photon counts and successfully demonstrated imaging of DNA origami samples with a feature distance of 12 nm. On this basis, MINFLUX fluorescence lifetime imaging is extended and realized by resolving a single fluosphere life signal from moving piezostage successively to configure a 7 nm-side-square.

To further reduce the system complexity, Luciano published RASTMIN recently^16^, which further simplifies the system and improves the multiplexing ability with other scanning-based microscopes. Here, an innovative single-molecule localization with sequential structured illumination^17^ (SML-SSI) method has recently been proposed. Based upon an inverted point-scanning confocal microscope, 200 ps-pulsed-640 nm excitation is modulated into a donut after the vortex-phase plate and a quarter-wave plate. The beam then passes through the lateral scanning part with dual-axis scanning on the sample plane with nm-level accuracy. The combined up-and-down drift-corrected paths (Fig. 1(a) gray part) then meet with excitation before the objective through a dichroic mirror (DM2). The lateral scan controls the donut excitation to illuminate the pre-determined square sample area (*L* ≈ 100 nm), which is close to TCP in MINFLUX and divided into K×K grid points. Scanning grid-by-grid is performed, whose structure seems like a raster, thus called RASTMIN (single-molecule localization by RASTer scanning a MINimum of light). The fluorescent signal obtained by traversing each grid is de-scanned by the xy-scanner, then enters the single-photon detector, and is ultimately analyzed by the time-correlated single-photon counter module. Each grid corresponds to a certain number of photon counts, forming an intensity image of K×K pixels. The acquired data is first screened for single-molecule emission on-state signals using the background threshold defined in the analytical model. Then, they compare it with a high signal-to-noise ratio image averaged by scanning a single fluorescent bead multiple times in advance, whose signal-to-background ratio (SBR) serves as the referee in (1)^17,18^, and use the maximum likelihood estimation to obtain the localization of a single molecule.

1 $${\rm SBR}\left( {{{{\boldsymbol{r}}}},L} \right) = \frac{{\mathop {\sum }\nolimits_{i = 1}^N I\left( {{{{\boldsymbol{r}}}} - {{{\boldsymbol{r}}}}_i} \right)}}{{\mathop {\sum }\nolimits_{i = 1}^N I\left( {0 - {{{\boldsymbol{r}}}}_i} \right)}}{\rm SBR}\left( {0,L} \right)$$

where SBR(***r***,*L*) is the signal-to-background ratio when the emitter at position ***r*** on the sample plane under the specific TCP range *L* (the emitter at the center of TCP ***r*** = 0 yields a benchmark), and *I*(***r****–****r***_*i*_) is the signal detected when the emitter at ***r*** with respect to excitation position ***r***_*i*_ (***r*** = 0 yields TCP center signal). Afterward, Monte Carlo simulation is used to define the confidence threshold for distinguishing single-molecule blinking or simultaneous blinking of multiple molecules to eliminate the emission events of multiple fluorescent molecules that do not obey the Poisson distribution. To achieve such a high positioning accuracy without ultra-high-speed photoelectric deflection devices, the expense is to configure a drift stabilization for a real-time offset correction of the excitation within 2 nm of the three dimensions in x-y-z in the sample plane (x, y < 1.2 nm; z < 2 nm). Stabilization is divided into two optical paths: excitation and detection. In the excitation path, the near-infrared 775 nm TM_00_ Gaussian mode laser is split into two light paths after passing through the beam splitter. The first path is a confocal excitation, which propagates through a pair of conjugate and tube lenses, expanding the beam and entering the back focal plane (BFP) of objective under parallel light. Thus, a convergent point on the sample excites and confocally scans the gold nanoparticles. The second beam is expanded by a telescope and then focused on the BFP of the objective. Thus, the widefield illumination is formed on the sample plane. The confocal detection consists of a pinhole placed on the Fourier surface of a pair of lenses in front of a camera to block the out-of-focus signals. Finally, the scattered signals from gold nanoparticles (GNs) were collected for imaging, as illustrated in Fig. 1(a). The system performs imaging tests on 2 × 3 DNA origami arrays with a wide interval of 20 nm and a length of 15 nm, achieving super-resolution imaging with a 2-nm localization precision. At the same time, the same single 40 nm dark red fluorophore is moved in 7 nm step along the xy direction to form a square by the translation stage, and the fluorescence lifetime imaging under 7 nm resolution was obtained by the time-correlated single photon counting module.

**Fig. 1:**
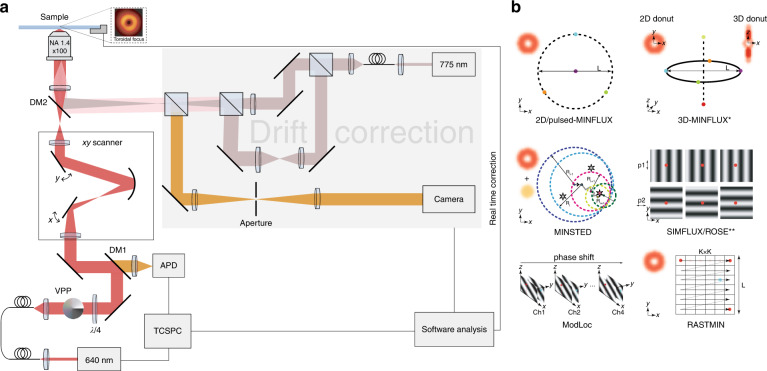
RASTMIN setup illustration and the main modulation patterns in some selected sequentially structured illumination(SSI)-based techniques. **a** RASTMIN setup consists of excitation with donut modulation, xy-scan, drift correction and time-related single photon detection modules which has high compatibility with scanning-based fluorescent microscope, if starting from the experience on STED construction. **b** Structured excitation in 2D-MINFLUX^13^, pulsed-MINFLUX^15^, 3D-MINFLUX^14^, MINSTED^21^, SIMFLUX^20^, ROSE^19^, ModLoc^22^, and RASTMIN^16^, respectively. ^*^Regular focus is not displayed. ^**^Only crucial coherent modulation is illustrated; the delicate control and difference are not displayed.

After the invention of MINFLUX, there were methods given birth that gather signals to localize single molecules interrogated with a specific sequence of spatial modulated excitation patterns such as Repetitive Optical Selective Exposure^19^(ROSE), SIMFLUX^20^, MINSTED^21^, and Modulation Localization Microscopy^22^(ModLoc); and even early exploring on molecular dipole orientation^23^. In Table 1, we compared some critical parameters, and structured excitation patterns (Fig. 1(b)) among the main sequential-structured-illumination (SSI) based localization microscopy techniques. Compared with utilizing random fluorescence fluctuation information in SOFI^24^, ROSE and SIMFLUX take advantage of active-modulated-induced fluctuation (coherent modulation) to attain localization information, and similarly the Bessel beam bestowed with axial gradient intensity^25^ yields another active fluorescence fluctuation localization for three-dimensional imaging. Clearly, non-ultrafast optoelectrical elements bring TCP pattern altering, specifically, the scanning configuration. In conventional MINFLUX, donut excitation is deflected sequentially to form a Star-like TCP. Meanwhile, it iteratively converges (dual-axis scanning with EODs up to 20 kHz), relying on the related position between the target and TCP itself until a single emitter localization process is finished. Comparatively, raster scanning reaches approximately 1 kHz without iterations and shrinks due to the limited speed and movement property of galvo mirrors. Another, the simplest RASTMIN achieves both similar localization precision and spatial resolution with only a set of core galvo-mirrors, piezo-stages and DAQ. The reason that RASTMIN achieves comparable performance to MINFLUX is because *σ*_*CRB*_ relays on the position of that molecule, the total detected photons for localization estimation, the signal-to-background ratio (SBR), and the size of the scanning area when the excitation intensity I(***r***) is assured. Since the detected photons obey Poisson distribution and the corresponding intensity on a single photon detector is linear with the photon counts from each pixel of K, in the likelihood function L^13,17^, appropriately increasing sampling numbers and/or total detected photons result in the higher localization precision and resolution.

**Table 1:** Core parameters, and core ultra-fast optoelectrical elements and control devices/software in main sequential structured illumination-based localization microscopies

	Localization precision (nm)	Spatial resolution (nm)	Scanning frequency (kHz)	Core ultra-fast optoelectrical elements and control devices/Software								
2D-MINFLUX^13^	~1	~6	8	AOTF	EOM	EOD		Tip/Tilt Piezo	Piezo stage	FPGA	DAQ	
3D-MINFLUX^14^	~1	1–3	20	AOTF	EOM	EOD		Tip/Tilt Piezo	Piezo stage	FPGA	DAQ	Varifocal lens
MINSTED^21^	1–3	1–3	125			EOD		Galvo mirrors	Piezo stage	FPGA		
SIMFLUX^20^	~10	<30	0.25				Pockels cell		Piezo stage	Arduino		
ROSE^19^	~2	<5	8	AOTF	EOM			Resonant mirror	Piezo stage	Complex programmable logic device	DAQ	
ModLoc^22^	~7	~12	1.2		EOM		Pockels cell			Four-channel trigger generator		
RASTMIN^16^	~1–2	~15	1					Galvo mirrors	Piezo stage	Adwin	DAQ	Time-correlated single-photon counting unit
p-MINFLUX^15^	~1–2	~12	100	Electromechanical shutters					Piezo stage	ADwin	DAQ	Time-correlated single-photon counting unit

To reduce the engineered barrier and achieve fast construction, such as super-precision systems, RASTMIN is also an inspiration. With the popularization of the STED system review on spatial and temporal resolution^26^ with the help of certain dyes^6,27^, moreover, the optically engineered STED protocol for self-building in its own lab^28^, the SSI-based localization system can quickly refit a prototype with performance close to MINFLUX for laboratories that have STED (especially stabilization is a commonly utilized path in observing GNs-samples’ sectioning PSF along both lateral and axial plane), or even more basically, confocal fluorescence microscope constructing experience. On this basis, do some avant-garde imaging tests or explorations, such as multi-photon multi-color MINFLUX^18^. Luciano et al. also theoretically predict the CRB localization precision and lateral symmetry of multiphoton RASTMIN^29^.

Although MINFLUX is termed the post-Noble-Prize-era super-resolution technique, there is unwanted signal crosstalk. Because of the shrinking scale of donut excitation, when there are two fluorescent molecules in the vicinity of single-molecule distance, the non-zero intensity along the donut ring undesirably excites the emitter far away from another close to the zero-center, thus emerging a stronger background^21^. That leads to the necessity for more complex and sophisticated models, and algorithms to eliminate the wrongly excited signals from the periphery. Further, it even needs the improvements in the MINFLUX TCP, for example, increasing scan points number along TCP for delicate samplings, hence coming out more accurate localization data. The problem comes from the intrinsic characteristic of the shrinking donut configuration. To this end, Michael. et al. invented MINSTED^21^ in 2021, using ONB-2SiR dye^30^ to label U-2 OS cells for imaging and addressing the legacy mentioned above problem. MINSTED is a new generation of super-resolution fluorescence microscope technique inspired and developed by the STED system, MINFLUX algorithm and novel anti-two-photon-absorption photoswitchable dyes. Due to its donut-raster scanning simplified from STED, this may pave a new and transformative avenue for developing RASTMIN for STED-based localization microscopy.

Combining specific modulated-point spread function (PSF) scanning confocal instruments and statistical estimation-based single-molecule localization algorithms has promoted super-resolution microscopy to a single nanometer. In the near future, it will lead to optical fluorescence nanoscopy to step into the Ångstrom era. We hope the booming towards both seeking for the magnitude limitation as human beings powerful tools to reclaim unknown wilderness, and simplifying the precision nanoscopy construction barrier to facilitate global collaboration for complex life science process exploration, can flourish more research fields and extensive investigations.
